# Treatment and prognosis of patients with late rectal bleeding after intensity-modulated radiation therapy for prostate cancer

**DOI:** 10.1186/1748-717X-7-87

**Published:** 2012-06-12

**Authors:** Shinya Takemoto, Yuta Shibamoto, Shiho Ayakawa, Aiko Nagai, Akihiro Hayashi, Hiroyuki Ogino, Fumiya Baba, Takeshi Yanagi, Chikao Sugie, Hiromi Kataoka, Mikio Mimura

**Affiliations:** 1 Department of Radiology, Nagoya City University Graduate School of Medical Sciences, Nagoya, 1 Kawasumi, Mizuho-cho, Mizuho-ku, Nagoya, 467-8601, Japan; 2 Department of Radiology, Nagoya Daini Red Cross Hospital, 2–9 Myoken-cho, Showa-ku, Nagoya, Aichi, 466-8650, Japan; 3 Department of Gastroenterology and Metabolism, Nagoya City University Graduate School of Medical Sciences, Nagoya, 1 Kawasumi, Mizuho-cho, Mizuho-ku, Nagoya, 467-8601, Japan

**Keywords:** IMRT, Radiation proctitis, Late toxicity

## Abstract

**Background:**

Radiation proctitis after intensity-modulated radiation therapy (IMRT) differs from that seen after pelvic irradiation in that this adverse event is a result of high-dose radiation to a very small area in the rectum. We evaluated the results of treatment for hemorrhagic proctitis after IMRT for prostate cancer.

**Methods:**

Between November 2004 and February 2010, 403 patients with prostate cancer were treated with IMRT at 2 institutions. Among these patients, 64 patients who developed late rectal bleeding were evaluated. Forty patients had received IMRT using a linear accelerator and 24 by tomotherapy. Their median age was 72 years. Each patient was assessed clinically and/or endoscopically. Depending on the severity, steroid suppositories or enemas were administered up to twice daily and Argon plasma coagulation (APC) was performed up to 3 times. Response to treatment was evaluated using the Rectal Bleeding Score (RBS), which is the sum of Frequency Score (graded from 1 to 3 by frequency of bleeding) and Amount Score (graded from 1 to 3 by amount of bleeding). Stoppage of bleeding over 3 months was scored as RBS 1.

**Results:**

The median follow-up period for treatment of rectal bleeding was 35 months (range, 12–69 months). Grade of bleeding was 1 in 31 patients, 2 in 26, and 3 in 7. Nineteen of 45 patients (42%) observed without treatment showed improvement and bleeding stopped in 17 (38%), although mean RBS did not change significantly. Eighteen of 29 patients (62%) treated with steroid suppositories or enemas showed improvement (mean RBS, from 4.1 ± 1.0 to 3.0 ± 1.8, *p* = 0.003) and bleeding stopped in 9 (31%). One patient treated with steroid enema 0.5-2 times a day for 12 months developed septic shock and died of multiple organ failure. All 12 patients treated with APC showed improvement (mean RBS, 4.7 ± 1.2 to 2.3 ± 1.4, *p* < 0.001) and bleeding stopped in 5 (42%).

**Conclusions:**

After adequate periods of observation, steroid suppositories/enemas are expected to be effective. However, short duration of administration with appropriate dosage should be appropriate. Even when patients have no response to pharmacotherapy, APC is effective.

## Background

Chronic rectal bleeding is one of the most common complications of radiation therapy for prostate cancer. The etiology of radiation proctitis is considered to be chronic mucosal ischemia caused by tissue fibrosis and obliterative endarteritis. This injured rectal wall can bleed easily, occasionally leading to a chronic ischemic state and causing episodes of severe rectal bleeding [1]. Several studies reported on the management of radiation proctitis after conventional pelvic radiotherapy. Traditional pharmacotherapy of hemorrhagic radiation proctitis includes anti-inflammatory agents, rectal steroids, rectal sucralfate, short-chain fatty acid enemas, and formalin therapy. Surgery is associated with high rates of morbidity in patients with previous radiation therapy and is therefore usually avoided [2]. Other treatment approaches have included hormonal therapy and hyperbaric oxygen. Endoscopic coagulation with a variety of devices, such as the yttrium-aluminum-garnet laser, heater and bipolar probes, and argon laser, has been reported to be effective for the control of bleeding. However, argon plasma coagulation (APC) is now established as an effective treatment for moderate or severe radiation proctitis, reducing rectal bleeding and iron or blood transfusion requirements by cauterizing mucosal telangiectasias [3]. In addition, few complications have been reported with the use of APC, with a reported rate of 2.5% compared with 5% to 15% for laser treatment [4].

Intensity-modulated radiation therapy (IMRT) is now commonly used in place of conventional irradiation in the treatment of localized prostatic cancer, and appears to have yielded higher local control rates than conventional radiotherapy [5-7]. In IMRT, radiation dose to the rectal wall is planned to be as low as possible, but radiation proctitis is still the most commonly encountered complication [8-11]. Radiation proctitis after IMRT seems to differ from that seen after whole-pelvic irradiation because this adverse event is a result of high-dose radiation to a very small area in the rectum. To our knowledge, treatment of late rectal complication after IMRT has not been reported systematically. We have used steroid suppository or enema for pharmacotherapy, and APC as an endoscopic therapy. The purpose of this study was to evaluate the results of these treatments for hemorrhagic proctitis after IMRT for prostate cancer.

## Methods

### Patients

Between November 2004 and February 2010, 403 patients with localized prostate cancer were treated with IMRT with or without hormone therapy at 2 institutions, Nagoya City University Hospital (NCUH) and Nagoya Daini Red Cross Hospital (NDRCH). Among these patients, 64 patients (16%) who developed late rectal bleeding were evaluated; 40 patients received IMRT using a linear accelerator at NCUH (linac group) and 24 patients by helical tomotherapy at NDRCH (tomotherapy group). The patient and tumor characteristics are shown in Table 1. Their median age was 72 years (range, 53–84); patients treated with tomotherapy were slightly older (p = 0.0036). D’Amico’s risk classification [12] was low in 14 patients, intermediate in 16, and high in 34. The American Joint Committee on Cancer clinical T stage was T1 in 22 patients, T2 in 25, and T3 in 17. There were more high-risk patients and T3 patients in the linac group than in the tomotherapy group. Forty-eight patients received hormone therapy; basically, neoadjuvant hormone therapy was performed in intermediate- and high-risk patients (according to D'Amico's classification) for 6–9 months (median, 7 months) and adjuvant hormone therapy was given to high-risk patients for 2–3 years (median, 28 months). Eighteen patients received antithrombotics before, during, and after IMRT for cardiovascular or cerebrovascular disease. The IMRT studies were performed prospectively with informed consent from all patients, but the present study evaluating rectal bleeding was a retrospective one.

**Table 1:** Patient characteristics

	**Total**	**Linac group**	**Tomotherapy group**
	n = 64	n = 40	n = 24
Median age	72	69	74
(range)	(53–84)	(55–79)	(53–84)
D'Amico's risk classification			
Low	14	6	8
Intermediate	16	9	7
High	34	25	9
Clinical T stage			
T1	22	12	10
T2	25	15	10
T3	17	13	4
Gleason sum score			
≤ 6	25	13	12
7	25	17	8
≥ 8	14	10	4
Initial PSA (ng/ml)			
< 10	31	18	13
10-20	18	10	8
> 20	15	12	3
Use of ADT	48	32	16
Use of antithrombotics	16	8	8
Presence of DM	10	4	6

ADT, androgen deprivation therapy; DM, diabetes mellitus.

Patients who developed late rectal bleeding were assessed clinically and/or endoscopically. Late rectal bleeding appeared no earlier than 3 months after the initiation of IMRT. The bleeding was graded using Common Toxicity Criteria for Adverse Events (CTCAE) version 4.0 and the Rectal Bleeding Score (RBS). This scoring system has been elaborated by ourselves for this study and is proposed to evaluate the grade of bleeding and efficacy of treatment. The RBS is the sum of Frequency Score and Amount Score. The Frequency Score was evaluated as follows: score 3, 3 or more episodes of bleeding per week; score 2, 0.5-2 episodes per week; and score 1, less than one episode in 2 weeks. The Amount Score was evaluated as follows: score 3, severe (reddened toilet bowl); score 2, moderate (blood on stool surface); and score 1, mild (blood spot on paper). When bleeding had stopped for over 3 months, RBS was recorded as 1. RBS was evaluated at the onset of bleeding and at the latest follow-up (> 12 months after bleeding onset).

### Intensity-modulated radiotherapy

All patients were immobilized in a supine position with a vacuum bag system for their whole body and CT scans were performed at a slice thickness of 3.2 mm. The clinical target volume (CTV) included the prostate and seminal vesicles (SV). The CTV of the SV depended on the T stage of the patient: 1/3 volume of the SV for T1; 1/2 of the SV for T2; and the whole SV for T3. The CTV was expanded in three dimensions with 6- to 8-mm margins to obtain the planning target volume (PTV). The rectum was contoured from the level 10 mm below the lower PTV edge or the anal verge (a higher one was chosen) to the level 10 mm above the upper PTV edge. In the linac group, IMRT was delivered with 18-MV photons of 5 static ports at 45°, 98°, 180°, 262°, and 315° using dynamic multileaf collimators. Eclipse Version 6.5/7.5 (Varian Medical Systems, Palo Alto, CA, USA) was used for dose calculations. In the tomotherapy group, IMRT was performed with 6-MV photons and the treatment planning system was Pinnacle3 (Philips Medical Systems, Madison, WI). The planned doses were 70 to 78 Gy (median, 76 Gy) delivered in 33 to 39 fractions (median, 37 fractions). The daily dose has been increased step by step from 2.0 Gy to 2.1 Gy, and more recently to 2.2 Gy. As dose-volume constraints for the rectum, < 35% and < 18% of the rectal volume were allowed to receive more than 51.3-51.5% and 76.9-77.3% (depending on the protocol), respectively, of the prescription dose. The maximum dose to the rectum was set at 100.0-100.4% of the prescription dose. The mean rectum volume was 59.0 ± 21.5 cc (median, 54.7 cc; range, 25.4-160.1 cc), the mean %V40Gy (% of rectum volume receiving ≥ 40 Gy) was 36.7 ± 7.1% (median, 35.0%; range, 25.8-60.8%), and the mean %V70Gy (% of rectum volume receiving ≥ 70 Gy) was 8.8 ± 5.4% (median, 8.1%; range, 1.3-30.4%).

### Treatment of rectal bleeding

Except for 19 patients with relatively severe bleeding, 45 patients were first observed without treatment for at least 3 months. A steroid suppository or a steroid enema was administered as pharmacotherapy in 29 patients; 18 received steroid as an initial therapy and 11 with poor improvement after observation over 3 months were given steroid treatment. One of the following suppositories was prescribed to 23 patients: Posterisan® Forte Ointment (Maruho Co., Ltd., Osaka, Japan; hydrocortisone 2.5 mg + Escherichia coli), Neriproct® Suppository (Bayer Yakuhin, Ltd., Osaka, Japan; diflucortolone valerate 0.2 mg + lidocaine 40 mg), and Rinderon® Suppository 1.0 mg (Shionogi & Co., Ltd., Osaka, Japan; betamethasone 1.0 mg). These suppositories were assumed to have similar efficacies, and choice of the suppositories was dependent upon the availability of the drugs at pharmacies of the patient’s preference. In the early stage of this study, Steronema® Enema 3 mg (Nichi-Iko Pharmaceutical Co., Ltd., Toyama, Japan; betamethasone sodium phosphate 3.95 mg as betamethasone 3 mg) was initially prescribed to 6 patients. In 1 patient, a steroid suppository was used at first but treatment was later changed to an enema because of ineffectiveness. Steroid enemas were more difficult to use for patients than steroid suppositories, and 2 patients could not undergo the enema successfully, so treatment was changed to a suppository immediately. Therefore, more recent patients were preferably treated with a steroid suppository. The frequency of steroid administration was once or twice daily, depending on the severity.

APC was used in 12 patients. It was used as an initial treatment in 1 patient with relatively severe bleeding, in 3 with poor improvement after observation over 3 months, and in 8 with poor response to the pharmacotherapy. The equipment used was a colonoscope (Olympus CF type H260, Tokyo, Japan), APC equipment with an APC probe with a diameter of 2.3 mm, an argon delivery unit, and a high-frequency unit (ERBE APC2, ERBE Elektromedizin GmbH, Tubingen, Germany). All patients were treated without sedation. The forced mode was used at an argon flow rate of 1.0 L/min with a power of 30–40 W. The frequency of administration was up to 3 times, depending on the severity.

### Statistical analysis

Mann-Whitney’s *U* test was used to evaluate difference in age between the linac and tomotherapy groups. Paired t-tests were used to compare the changes after treatments. The response rate over time of respective strategies was calculated by the Kaplan-Meier method; patients who showed no response and moved to the next treatment were censored at that time. Log-rank tests were used to examine the other clinical variables including radiotherapy technique (linac vs. tomotherapy), use of androgen deprivation therapy, use of antithrombotics, presence of diabetes mellitus (DM), rectal %V40Gy (≥ or < median), and rectal %V70Gy (≥ or < median). Statistical analyses were performed using the statistical software StatView Version 5 (SAS Institute Inc., Cary, NC). A p < 0.05 significance level (2-sided) was applied for all statistical tests.

## Results

**Figure 1:**
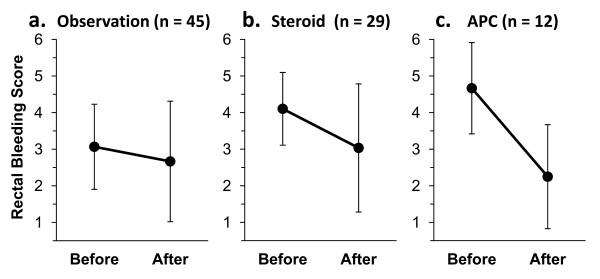
**(a, b, c): Changes in Rectal Bleeding Score (RBS) in the observation (a), steroid (b), and APC groups (c).** Solid spots and error bars represent the mean and standard deviation of the data.

**Figure 2:**
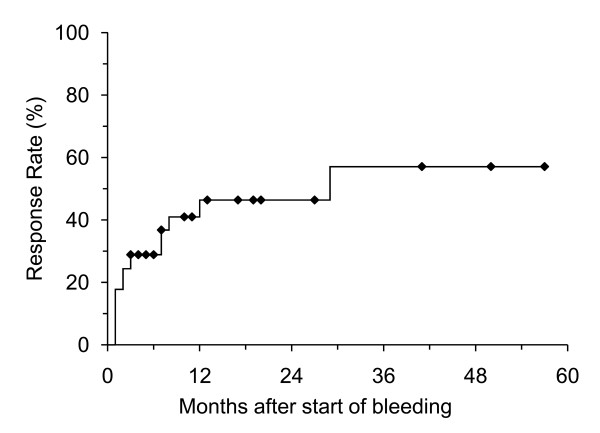
Kaplan-Meier curve for response in the observation group (n = 45).

The median follow-up period was 35 months (range, 12–69 months). CTCAE grade of rectal bleeding was 1 in 31 patients (48%), 2 in 26 (41%), and 3 in 7 (11%). Rectal bleeding occurred at 3–41 months (median, 13 months) after the initiation of IMRT. Forty-five patients were observed without treatment for at least 3 months, including patients who were administered steroid or APC after the observation period. Nineteen patients (42%) showed improvement and 4 showed exacerbations. The mean RBS changed from 3.1 ± 1.2 to 2.7 ± 1.6 (p = 0.25) and bleeding stopped in 17 patients (38%) (Figure 1a). The response rate was 29% at 6 months and 46% at 1 and 2 years (Figure 2). Fourteen patients (31%) are still being observed with a small amount of bleeding.

**Figure 3:**
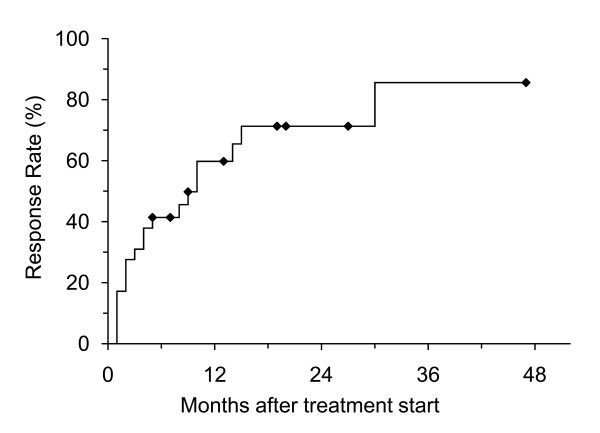
Kaplan-Meier curve for response in the steroid group (n = 29).

Twenty-nine patients received steroid suppositories and/or enemas; 18 (62%) showed improvement and 3 showed exacerbations. The mean RBS improved significantly from 4.1 + 1.0 to 3.0 + 1.8 (p = 0.003) and bleeding stopped in 9 patients (31%) (Figure 1b). The response rate was 41% at 6 months, 60% at 1 year, and 71% at 2 years (Figure 3). There was no significant difference in response rate between steroid suppository and steroid enema groups. All but one patient had no complication with steroid therapy; one patient developed septic shock and died of multiple organ failure after treatment with steroid enema 0.5-2 times a day for 12 months. Long-term rectal bleeding and/or prolonged use of steroid was considered to be a possible cause of death.

All of 12 patients treated with APC (Figure 1c) showed improvement within 2 months (mean RBS, from 4.7 ± 1.2 to 2.3 ± 1.4, p < 0.001) and bleeding stopped in 5 patients (42%). However, 2 or 3 sessions of treatment were needed to stop or relieve bleeding in 5 patients (42%). No patient developed complications requiring treatment.

Furthermore, we examined factors related to response rates after observation (Table 2) and steroid therapy (Table 3). Upon observation, the tomotherapy-group patients and patients with a rectal %V70Gy below the median (8.1%) had a better response rate at 1 year (p = 0.009 and 0.005, respectively). DM patients responded early to steroid therapy (p = 0.02). Other factors were not associated with response after observation or steroid therapy.

**Table 2:** Log-rank test for factors associated with improvement after observation.

**Factors**	**Groups (number of patients)**	**Response rate at 1 year (%)**	***P* value**
Age	≥ 71 (25)/≤ 70 (20)	54/35	0.12
IMRT method	Linac (30)/Tomotherapy (15)	20/100	0.009
Use of ADT	Yes (35)/No (10)	44/17	0.56
Use of antithrombotics	Yes (26)/No (14)	61/49	0.66
Presence of DM	Yes (6)/No (38)	67/42	0.37
Rectal %V40Gy	≥ 35.0% (27)/< 35.0% (17)	50/35	0.80
Rectal %V70Gy	≥ 8.1% (25)/< 8.1% (19)	33/65	0.005

ADT, androgen deprivation therapy; DM, diabetes mellitus. Rectal %V40/%70 Gy, % of rectum volume receiving ≥ 40 or 70 Gy.

**Table 3:** Log-rank test for factors associated with the response to steroid therapy.

**Factors**	**Groups (number of patients)**	**Response rate at 1 year (%)**	***P* value**
Age	≥ 71 (17)/≤ 70 (12)	66/51	0.85
IMRT method	Linac (16)/Tomotherapy (13)	46/72	0.33
Use of ADT	Yes (21)/No (8)	61/63	0.93
Use of antithrombotics	Yes (9)/No (15)	83/49	0.37
Presence of DM	Yes (7)/No (22)	100/44	0.02
Rectal %V40Gy	≥ 35.0% (14)/< 35.0 % (15)	50/83	0.27
Rectal %V70Gy	≥ 8.1 % (15)/< 8.1 % (14)	47/85	0.18

ADT, androgen deprivation therapy; DM, diabetes mellitus; Rectal %V40/%70 Gy, % of rectum volume receiving ≥ 40 or 70 Gy.

## Discussion

The development of adverse events related to radiation therapy depends on the dose and volume of normal tissues irradiated [13]. Generally, doses exceeding 50 Gy increase the potential of radiation damage, and the incidence could be as high as 30% when the radiation dose exceeds 70 Gy [14]. In most patients who had an endoscopic examination in the present study, a relatively small region of proctitis was detected, which was thought to represent adverse events of IMRT delivering a high dose to a limited area of the rectum. In our recent analysis, however, the incidence of radiation proctitis was correlated with %V40Gy of the rectum rather than %V60Gy or %V70Gy (A. Hayashi et al., manuscript in preparation).

Patients observed for certain periods without treatment showed slight improvement of the bleeding score but this improvement was not statistically significant. Since bleeding stopped spontaneously in 38% of the patients, we think that patients with late rectal bleeding should be observed for 3 to 6 months unless their symptoms are severe or become exacerbated during the follow-up period. In other studies too, most of the patients showed improvement of endoscopic changes after prostate radiotherapy without treatment [15,16]. Obviously, when patients show severe bleeding leading to decrease of the hemoglobin level, immediate treatment with steroids or APC is recommended. Patients who had been treated by tomotherapy showed earlier response, but this is thought to be due to a bias in patient populations between the 2 institutes: the linac group had more T3 patients (33%) than the tomotherapy group (17%). The larger CTV for the SV due to the advanced T stage might have caused worse adverse events in the linac-group patients. The smaller rectal %V70Gy was associated with a better response rate after observation, while the %V40Gy was not. This result appears quite reasonable, but does not agree with the above-mentioned result that %V40Gy was correlated with the incidence of rectal bleeding. We will further investigate the issue with more patients and longer follow-up periods.

Corticosteroids exert their anti-inflammatory effects in part by inhibiting histamine release and thereby stabilizing mast cells. It is anticipated that corticosteroids will help alleviate the symptoms of radiation proctitis. The efficacy of steroid enemas in the management of chronic radiation proctitis has yet to be proven, although slight symptomatic alleviation was proven in patients treated with steroid enemas and oral sulfasalazine in a small prospective double-blind study [17,18]. Intestinal bleeding after pelvic radiation therapy may be somewhat difficult to treat with steroid because radiation proctitis or colitis is more widespread. We used not only steroid enemas but also suppositories, in the expectation that the suppositories could be effective for small regions of proctitis after IMRT located just above the anal canal. There have been some studies on pharmacotherapy of rectal bleeding [19,20], but no report exists regarding proctitis after IMRT, to the best of our knowledge. Overall, patients showed a significant improvement by steroid suppository/enema therapy and bleeding stopped in 31% in the present study. Therefore, steroid suppositories and enemas seem to be worthy of consideration when bleeding does not stop after observation. However, short duration of administration with appropriate dosage should be appropriate because long-term, high-dose, frequent administration of steroid suppositories/enemas could cause several adverse effects including rounding of the face (moon face), high blood pressure, exacerbation of DM, and increased susceptibility to infection [21]. Rectal steroids may show toxicity since their systemic bioavailability is up to 44% of that of an equivalent oral dose [22]. Earlier response was observed in patients with DM. We have no explanation for this observation and it might be related to the small number and biased selection of patients for the Kaplan-Meier method. In one study, patients treated with antithrombotics more often developed grade 2 or 3 late rectal toxicity after external beam radiotherapy [23], but responses to treatments were not investigated. We could not find any differences in the response rates with or without antithrombotics.

APC uses the ‘plasma’ of ionized argon gas, which heats the mucosal surface to a depth of 0.5 to 3 mm, coagulating the superficial blood vessels [24]. Several studies reported that APC successfully ameliorated rectal bleeding associated with hemorrhagic radiation proctitis in 76-100% of cases [3,25,26]. Each study used a different argon flow rate and voltage, and complications also differed, including severe ones such as rectal strictures and perforations. Although there are no clear guidelines of precise APC settings for radiation proctitis, it was reported that the argon rate should be set at 1.0-1.5 L/min and a power of 40–50 W should be used [24]. The power can be increased to 60 W for areas of significant hemorrhage. Treatment is concentrated on the area of most prominent telangiectasia, leaving areas of untreated mucosa in between. Single or repeat pulses of less than 1 second are used, but care should be taken not to overlap or treat a particular area of rectal mucosa repeatedly; otherwise this increases the risk for mucosal ulceration that is characteristically slow to heal. Chino et al. [27] also reported that low-power (40 W) and short-time (1–2 sec) settings provided sufficient effect with low risk. However, some patients who have severe proctitis, such as dilated veins associated with ulcers and erosions, showed serious complications. In these cases, APC is unlikely to be successful although it may ameliorate symptoms to some extent. We think that APC is effective especially in proctitis following IMRT since almost all of our patients had proctitis in so small areas of the rectum that complications could be reduced. In our study, bleeding stopped in 42% and no patients showed serious complications after APC.

## Conclusions

To manage rectal bleeding that does not disappear after adequate periods of observation, a steroid suppository or enema is expected to be effective and easy to use for patients. Even when patients have no response to pharmacotherapy, APC is effective and stops or decreases bleeding in a relatively short period.

## Competing interests

The authors declare that they have no competing interests.

## Authors’ contributions

ST, TS, SA, AN, AH, HO, FB, TY, CS, HK, and MM. all authors have read and approved the final manuscript.
